# Benign Giant Phyllodes Tumours Presenting as Fungating Breast Masses

**DOI:** 10.7759/cureus.68589

**Published:** 2024-09-03

**Authors:** Karthik Deeban, Athira Gopinathan, Arun Bharathi, Nandhini G

**Affiliations:** 1 General Surgery, Sri Ramaswamy Memorial (SRM) Medical College Hospital and Research Centre, Chengalpattu, IND

**Keywords:** phyllodes tumours, ulceration, fungating tumour, newer treatment modality, giant

## Abstract

Breast masses presenting as fungating growths usually represent advanced malignancy. One remarkable exception is a benign phyllodes tumour. These tumours of stromal origin often exhibit rapid growth, resulting in pressure necrosis at the summit of the tumor causing fungation. It is difficult to differentiate between benign and malignant types clinically. Here, we describe two cases in which patients presented with fungating growth similar to a case of carcinoma breast, which turned out to be cases of benign phyllodes tumours. We would like to highlight the clinical features that differentiate between benign and malignant fungating growth and provide a brief update on the latest treatment modalities for phyllodes tumours.

## Introduction

Phyllodes tumour is a rare fibroepithelial tumour of the breast with a varied clinical presentation that accounts for 0.3%-0.5% of all primary breast neoplasm [1]. Previously known as cystosarcoma phyllodes, the current terminology "phyllodes tumour" is coined by the World Health Organisation [2] to highlight the pathological hallmark of the leaf-like pattern of the tumour. Although it occurs sporadically in middle-aged females, genetic conditions such as Li-Fraumeni syndrome and deletion of mutations of *BRCA1*, *BRCA2*, *RB1* and *TP53* genes may also play a role in its occurrence [3]. Phyllodes tumours of size more than 10 cm are called giant phyllodes tumours [4]. Phyllodes tumours can be classified into benign, borderline and malignant, based on histopathology. Prognosis and treatment vary with the type of tumour. Investigations such as fine needle aspiration cytology (FNAC) and core needle biopsy will guide in diagnosis, but histopathological examination will yield an exact diagnosis. Surgery (wide local excision) is the mainstay of treatment in both benign and malignant conditions. Borderline and malignant conditions are treated with a 1 cm margin [3]. It is known for its tendency for local recurrence. Adjuvant therapies are offered only in selected situations. Here, we report two cases of benign phyllodes tumour presenting as fungating masses.

## Case presentation

Case 1

**Figure 1:**
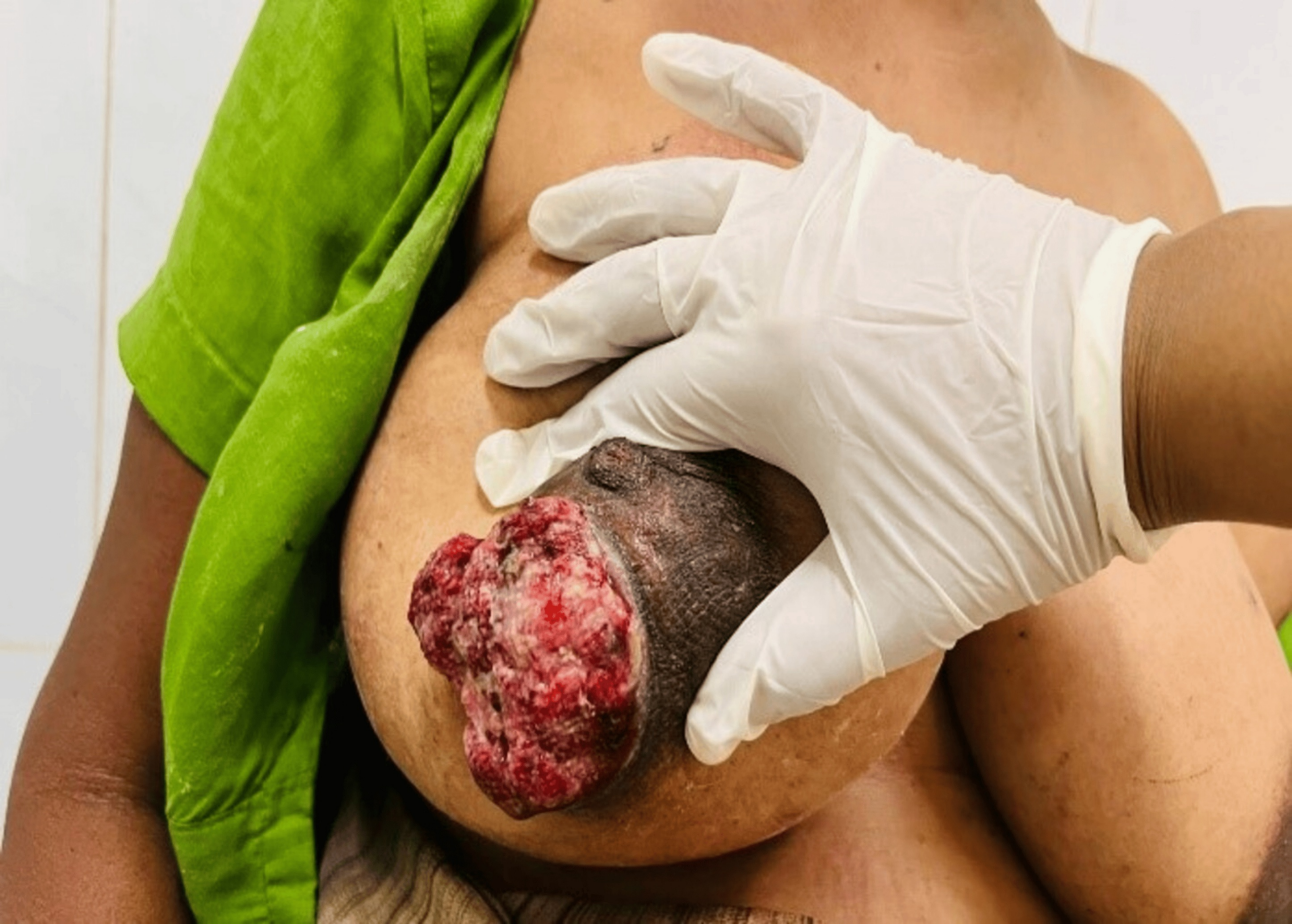
Fungating tumour of the right breast (case 1)

A 35-year-old female presented with a fungating mass in the right breast for the past month. She noticed a lump in the right breast for almost one year, which she ignored. She sought medical intervention when the lump exhibited rapid growth and caused fungation and ulceration. The patient reported only mild pain and oozing from the ulcer surface. Local examination of the right breast revealed a 3 × 3 cm fungating ulcer at the 6 to 9 o'clock position at the junction of the nipple-areolar complex (NAC) and skin (Figure 1). The ulcer had a regular surface with well-defined margins. The NAC was stretched out, but the nipple was intact. A 13 × 10 cm underlying lump was present involving the right breast predominantly in the outer lower quadrant under the NAC and was not fixed to the chest wall. No induration of the margins was present. The tumour was not infiltrating the skin margins on probing (overhanging edges). Ulcer bled upon touch. Right axillary discrete lymph nodes (central group) of size less than 1 cm and 2-3 in number were palpable. The opposite side breast and axilla were normal.

Investigations

**Figure 2:**
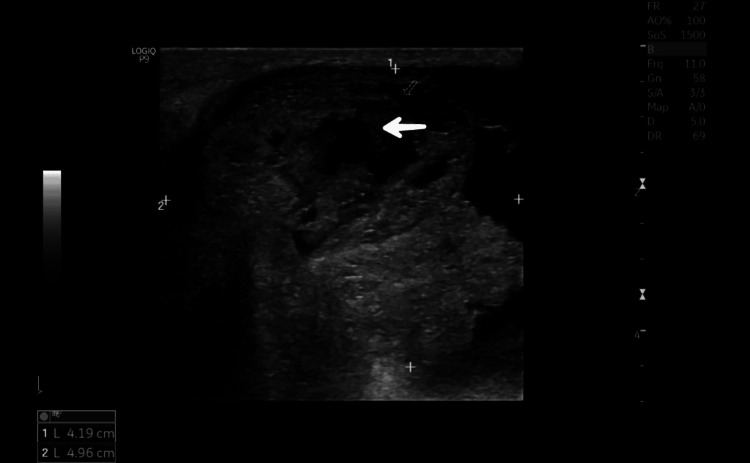
Breast ultrasound image of case 1 Ultrasound of the breast shows a large cystic lesion with solid components with free-floating internal echoes and internal vascularity (arrow) (BI-RADS IV C/V). BI-RADS: Breast Imaging Reporting and Data System

**Figure 3:**
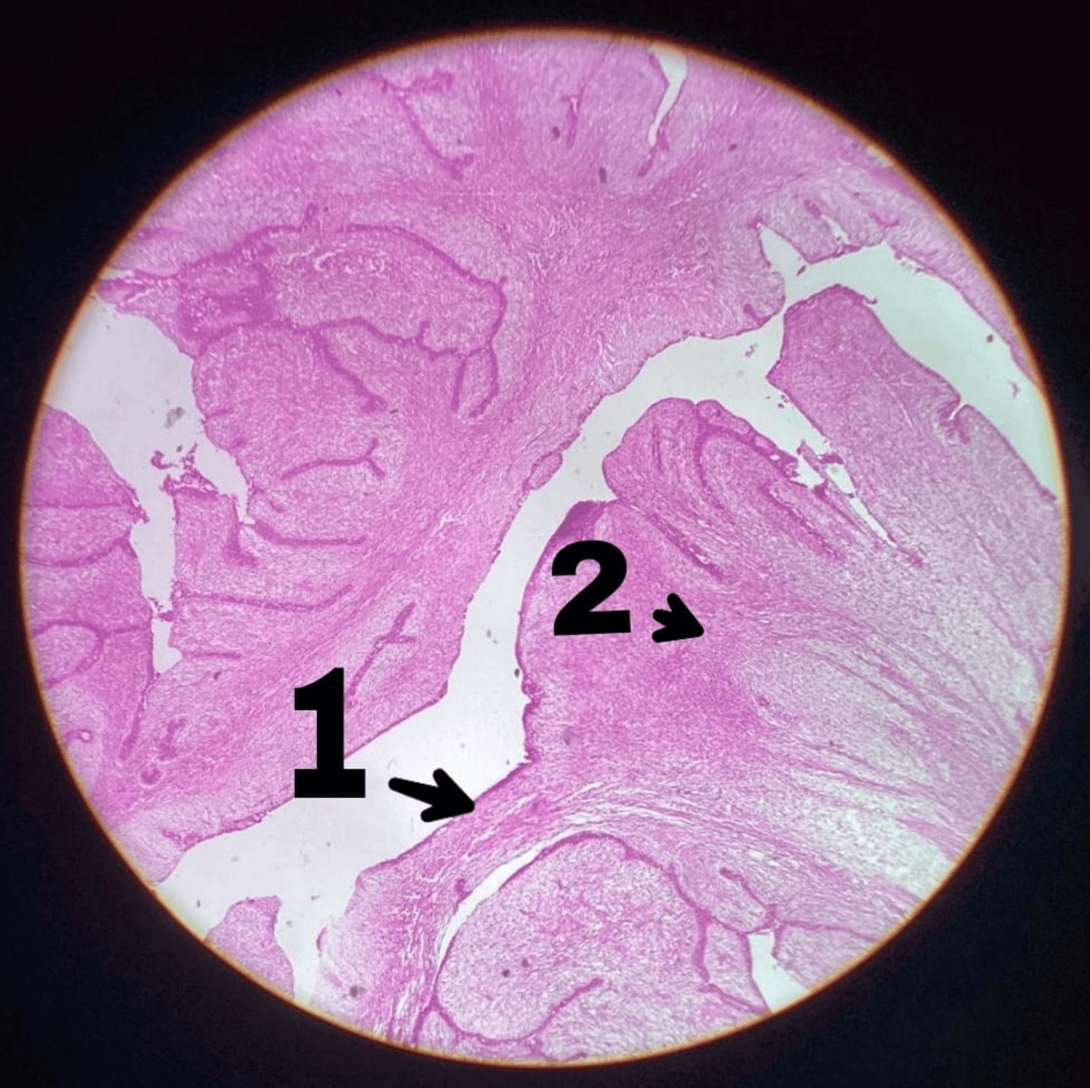
Histopathological image of case 1 1 indicates a benign phyllodes tumour with the characteristic leaf-like epithelial pattern. 2 indicates stromal cells in the benign phyllodes tumour showing minimal atypia, relatively low cellularity and inconspicuous mitosis.

Ultrasound of the breast showed a large cystic lesion with solid components and was reported as Breast Imaging Reporting and Data System (BI-RADS) IV C/V (Figure 2). Core needle biopsy showed features suggestive of benign phyllodes tumour. Right axillary lymphadenopathy (9 mm) was present. FNAC from the lymph nodes showed reactive changes. CT of the chest was normal. The patient underwent a simple mastectomy due to ulceration and because the huge lump was occupying most of the breast. Post-mastectomy histopathology report confirmed the benign nature of the phyllodes tumour (Figure 3).

Case 2

**Figure 4:**
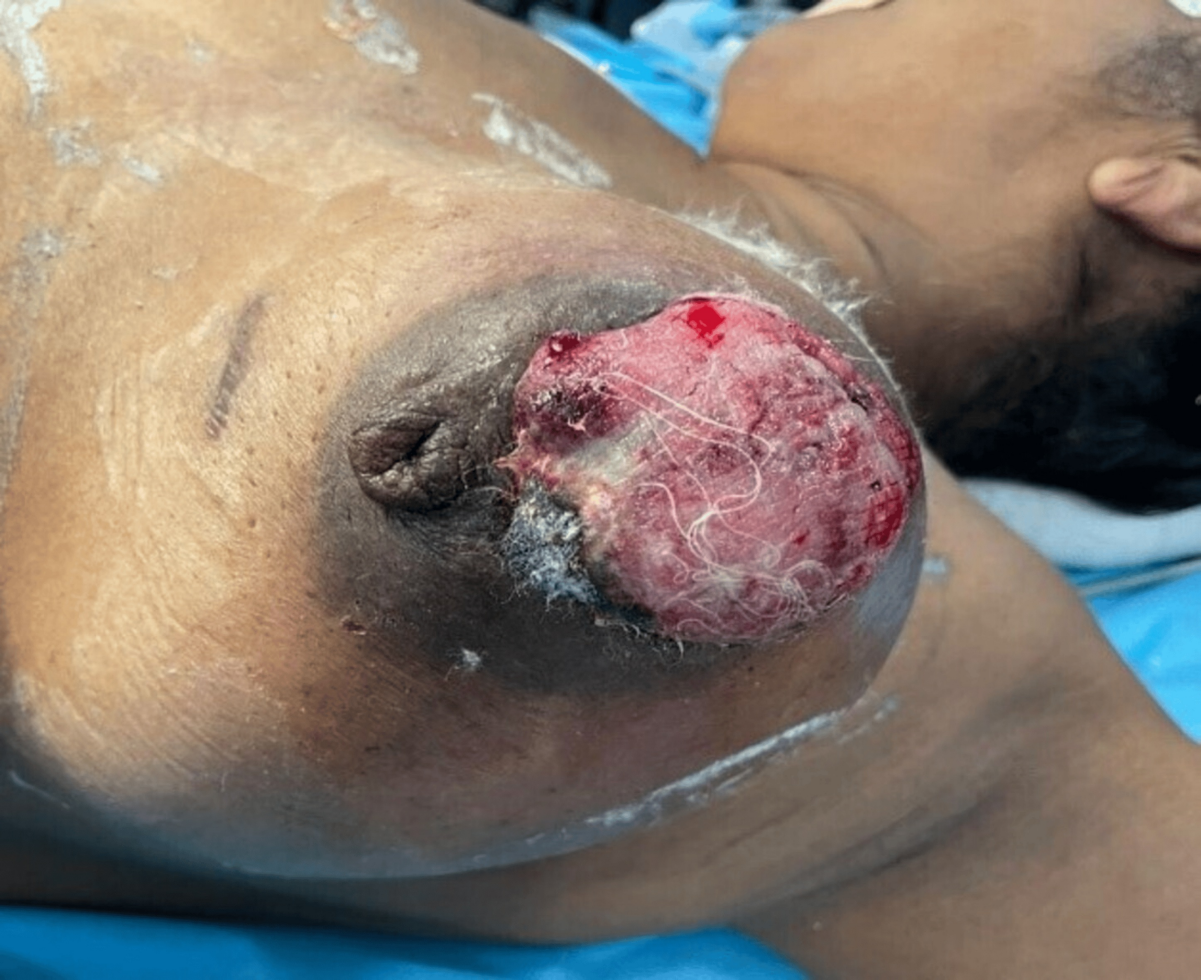
Fungating tumour of the left breast (case 2)

 A 48-year-old female patient presented with an ulcer in the left breast for the past two months. She had a lump in the left breast for the past 10 months. She had a history of incision and drainage for a left breast abscess done two months back. Local examination of the left breast revealed a 4 × 4 cm fungating ulcer over the breast between the 9 and 12 o'clock position involving the NAC (Figure 4). The ulcer had an irregular surface but regular margins with overhanging edges. There was no induration of margin and the ulcer bled upon touch. There was a lump measuring 15 × 11 cm occupying the entire breast under the ulcer. There was no chest wall fixity, nipple discharge or disfigurement. Two discrete axillary lymph nodes of size 1 cm were present in the left central group of lymph nodes. The opposite breast and axilla were normal.

Investigations

**Figure 5:**
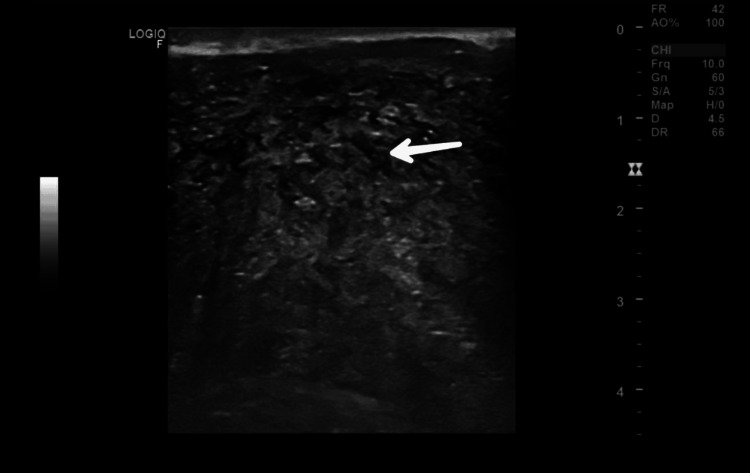
Breast ultrasound image of case 2 Ultrasound of the breast shows a large, relatively well-defined mass lesion involving all quadrants (arrow) (BI-RADS IV). BI-RADS: Breast Imaging Reporting and Data System

**Figure 6:**
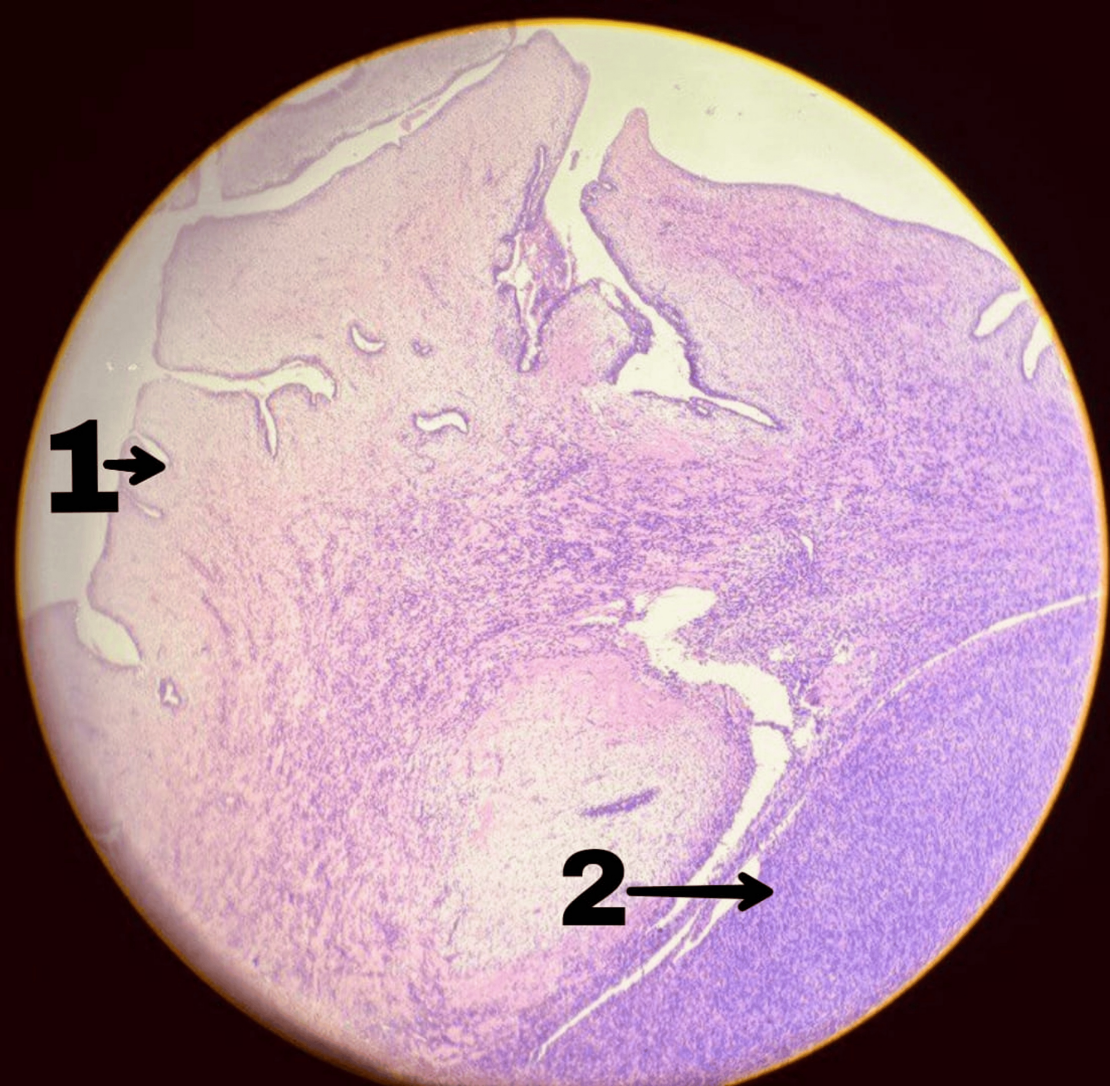
Histopathological image of case 2 1 indicates a benign phyllodes tumour with the characteristic leaf-like epithelial pattern. 2 indicates stromal cells in a benign phyllodes tumour showing minimal atypia, relatively low cellularity and inconspicuous mitosis (<5/10 hpf). hpf: high-power field

Ultrasound of the breast showed a large well-defined lesion graded as BI-RADS IV (Figure 5). Fine needle aspiration cytology and core needle biopsy suggested benign features with few cells showing mild atypia. CT of the chest was normal. The patient underwent a simple mastectomy due to the presence of an ulcer and tumour occupying the entire breast. The histopathology report confirmed a benign phyllodes tumour (Figure 6).

## Discussion

The initial presentation of these rapidly growing tumours arouses suspicion of carcinoma of the breast because of fungation, ulceration, contact bleeding and the presence of axillary lymph nodes. A close differential diagnosis is sarcoma of the breast. However, several points favoured the benign nature of the swelling such as the absence of chest wall fixity, regular margins, the absence of induration at the margins, lack of infiltration of the margin of swelling (overhanging edges) and the presence of intact nipple. According to the patient, the ulceration started at the junction of the nipple-areolar complex (NAC) and normal skin. This area may be the weakest area in the breast, which yields to the pressure of rapidly growing tumours and causes pressure necrosis and ulceration of the skin. In the case of malignancy of the breast, ulceration is caused by the infiltration of the tumour into the skin and does not necessarily occur at the junction of the NAC and skin. Benign phyllodes tumour might mimic a giant fibroadenoma, but patient age of around 35-55 years [5], size > 3 cm at presentation [6], rapid growth and a high chance of local recurrence favours phyllodes tumour.

Our cases fall into the category of giant phyllodes tumour. In our extensive literature research in PubMed, Google Scholar and others, we have come across only two case reports of benign phyllodes tumours presenting as fungating ulcers [7-9]. Giant phyllodes tumour have a high chance of malignancy (42.5%) compared to small benign phyllodes tumours (21%) and have a potential of tumour penetration into surrounding tissues [10]. Hence, it is recommended not only to remove the visible tumour but also the surrounding 1 cm rim of normal tissues to prevent recurrence. Both our patients underwent a simple mastectomy one year back, were under close follow-up and show no signs of recurrence till now [11]. Histopathology shows whorled stroma with large clefts, which resembles a leaf-like pattern.

Based on histopathological features such as stromal atypia, stromal cellularity, stromal overgrowth, mitotic counts and invading or infiltrating tumour borders, phyllodes tumour is classified into benign, borderline and malignant [12]. Ultrasound of the breast shows a well-demarcated, smooth, wider-than-tall hypoechoic mass. A mammogram may show a mass with a lobulated margin, and MRI suggests leaf-like solid segments in blood-filled cystic spaces. The sensitivity of FNAC is around 63% in establishing a diagnosis of a phyllodes tumour [13], and the sensitivity of core needle biopsy for a benign tumour is 64.2%, borderline tumour is 40% and a malignant phyllodes tumour is 100% [14]. Hence, surgical excision and histopathology are required to establish a final diagnosis.

Management depends on the histopathological type. Benign tumors are usually removed via enucleation/excision, and borderline and malignant cases should be managed based on National Comprehensive Cancer Network (NCCN) guidelines. It recommends borderline and malignant phyllodes tumours to be excised with a margin of more than or equal to 1 cm to prevent local recurrence [15]. Malignant phyllodes tumour usually spreads hematogenously; hence, lymph node excision is not recommended [16]. Post-operative radiotherapy has not much role but is used in cases of inadequate margins in borderline and malignant cases to prevent chest wall recurrence. Systemic therapy plays a limited role and is used in the palliation of metastatic disease. Agents such as ifosfamide, doxorubicin, cisplatin and etoposide are commonly used [17]. The use of newer agents such as tyrosine kinase inhibitors is also under study.

Benign cases have excellent prognosis and have very low chances of local recurrence. The chance of recurrence is high in younger women under 25 years of age, and these patients should be followed up with ultrasonography (USG) every six months for the next three years. Benign cases have a recurrence rate of 4% [18]. Metastatic lesions have a poor prognosis. The overall rate of metastasis is 4% for all phyllodes tumours [19]. Histopathological features such as stromal atypia, stromal overgrowth, infiltrative borders, increased mitotic activity of more than 10/10 hpf and positive margins post-surgery have a high chance of metastasis [19]. The usual sites of metastasis are the lungs, bones, liver and brain [20]. In these cases, overall survival is poor, usually less than three years.

## Conclusions

Our case report shows that all large, fungating tumours of the breast need not be malignant. Clinical features such as a rapidly growing tumour stretching the NAC but with an intact nipple, an overlying ulcer with regular margins, lack of chest wall fixity and overhanging edges (lack of infiltration) point to the benign nature of the disease. The breakout point at the junction of the NAC and skin in case of pressure necrosis needs to be validated with a larger case series. Histopathological examination and careful clinical examination will help in the diagnosis of a phyllodes tumour. It has a high risk of local recurrence; hence, adequate surgical margin is essential to offer a cure. Adjuvant treatment options have a limited role.
